# Dorsal turning of motor corticospinal axons at the pyramidal decussation requires plexin signaling

**DOI:** 10.1186/1749-8104-3-21

**Published:** 2008-08-26

**Authors:** Regina L Faulkner, Lawrence K Low, Xiao-Bo Liu, Jeffrey Coble, Edward G Jones, Hwai-Jong Cheng

**Affiliations:** 1 Center for Neuroscience, University of California, Davis, California, 95618, USA; 2 Department of Cell Biology and Human Anatomy, University of California, Davis, California, 95616, USA; 3 Department of Psychiatry and Behavioral Sciences, University of California, Davis, California, 95616, USA; 4 Department of Neurobiology, Physiology, and Behavior, University of California, Davis, California, 95616, USA; 5 Department of Pathology and Laboratory Medicine, University of California, Davis, California, 95616, USA

## Abstract

**Background:**

The development of the corticospinal tract (CST) in higher vertebrates relies on a series of axon guidance decisions along its long projection pathway. Several guidance molecules are known to be involved at various decision points to regulate the projection of CST axons. However, previous analyses of the CST guidance defects in mutant mice lacking these molecules have suggested that there are other molecules involved in CST axon guidance that are yet to be identified. In this study, we investigate the role of plexin signaling in the guidance of motor CST axons *in vivo*.

**Results:**

Expression pattern studies show that *plexin-A3*, *plexin-A4*, and *neuropilin-1 *are expressed in the developing cerebral cortex when the motor CST axons originating from layer V cortical neurons are guided down to the spinal cord. By analyzing mutant mice, we show that motor CST axons that turn dorsally to cross the midline at the pyramidal decussation require plexin-A3 and plexin-A4 signaling. Although other CST guidance defects are found in neuropilin-1 mutants, this dorsal turning defect is not observed in either neuropilin-1 or neuropilin-2 mutants, suggesting that the local cues that activate plexin signaling at the dorsal turning point are membrane-bound semaphorins. Further expression pattern study and mutant analysis indicate that Sema6A is one of the local cues for motor CST axon turning at the pyramidal decussation.

**Conclusion:**

Dorsal turning and midline crossing at the pyramidal decussation is a crucial step to properly direct CST axons into the dorsal spinal cord. We show that the signaling of plexin-A3, plexin-A4, and Sema6A is at least partially required for dorsal turning of the CST axons, while neuropilin-1 is required for proper fasciculation of the tract at midline crossing. Together with previous reports, these results demonstrate that several guidance cues are specifically utilized to regulate the dorsal turning and midline crossing of developing CST axons.

## Background

**Figure 1:**
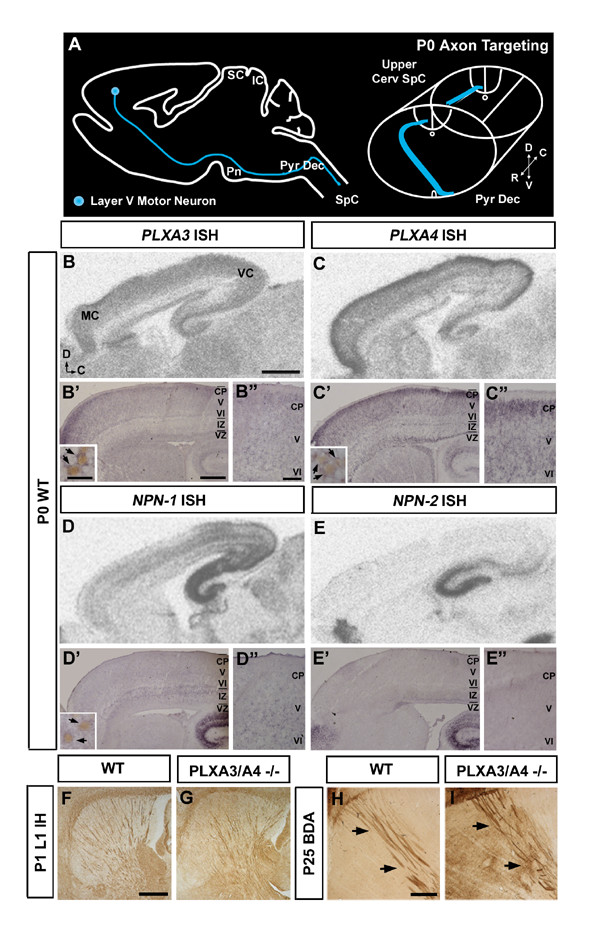
**Expression of *PLXA3*, *PLXA4*, *NPN*-*1*, and *NPN*-*2 *in the neocortex during corticospinal tract targeting.(a) **Diagram of sagittal view of the brain and cross-section of the brainstem and spinal cord representing axon targeting of the corticospinal tract at P0. **(b-e) ***In situ *hybridization of *PLXA3*, *PLXA4*, *NPN*-*1*, and *NPN*-*2*. Radioactive (b, c) and non-radioactive (b', b", c', c") *in situ *hybridization demonstrates that *PLXA3 *and *PLXA4 *mRNA is expressed throughout the neocortex at P0. *NPN*-*1 *mRNA (d-d") is expressed in deeper layers of the neocortex at P0. Insets in (b'-d') show cortical neurons (arrows) that co-express *PLXA3*, *PLXA4*, or *NPN*-*1 *with the layer V neuronal marker Ctip2. *NPN*-*2 *mRNA (e-e") is not expressed in cortex at P0. **(f, g) **L1 immunohistochemistry (IH) of the sagittal brain demonstrating the normal course of subcortical projections through the internal capsule of P1 WT and PLXA3/PLXA4-/- mice. **(h, i) **Sagittal sections of the brain showing the normal course of BDA-labeled subcortical projections from the motor cortex of P25 WT and PLXA3/PLXA4-/- mice. Black arrows indicate BDA-labeled axons descending through the internal capsule. C, caudal; CP, cortical plate; D, dorsal; IC, inferior colliculus; IZ, intermediate zone; MC, motor cortex; Pn, pons; Pyr Dec, pyramidal decussation; R, rostral; SC, superior colliculus; SpC, spinal cord; V, ventral; VC, visual cortex; VZ, ventricular zone. Scale bars: 1,000 μm (b-e); 400 μm (b'-e'); 25 μm (insets in b'-d'); 100 μm (b"-e"); 500 μm (f-i).

The formation of functional neural circuits within the central nervous system (CNS) requires proper guidance of axonal projections to specific target regions. The guidance of axons to distant targets within the CNS relies on the presence of signals at different choice points to guide axons along a correct pathway [1-3]. The corticospinal tract (CST) represents the longest projection pathway in the CNS of higher vertebrates [4-8]. In developing rodents, the CST axons originate from layer V cortical pyramidal neurons [7]. They exit the neocortex through the internal capsule and cerebral peduncle. In the brainstem, they are guided along the pyramidal tract and turn dorsally at the pyramidal decussation to cross the midline and reach the contralateral side of the spinal cord (Figure 1a). The targeting of primary CST axons to the spinal cord is followed by axon collateral branching to several target areas and then by pruning of specific collateral branches [7,9].

Recent evidence has demonstrated that molecules involved in axon guidance elsewhere in the CNS are also involved in regulating axon guidance decisions made by the CST [10]. Guidance of initial corticofugal projections to the cerebral peduncles is dependent on Slit function [11]. When CST axons approach the pyramidal decussation at the caudal medulla, intact netrin signaling via DCC and Unc5h3 receptors is required to prevent axon mistargeting [12]. The immunoglobulin (Ig) superfamily molecules L1 and NCAM have been implicated in maintaining the fidelity of the CST bundle as it turns and crosses at the pyramidal decussation [13,14]. As CST axons travel caudally from the decussation, repulsive cues by Wnt morphogens seem to determine the rostro-caudal positioning of the axons in the dorsal columns of the spinal cord [15]. Finally, when CST axons collateralize within the contralateral gray matter of the spinal cord, ephrin signaling is required to prevent axon branches from re-crossing the midline [16,17]. Together, the evidence demonstrates that the guidance choices of CST axons are highly dependent on the presence of local cues in their CNS environment. However, since loss of these molecules only results in partial defects in CST targeting, additional axon guidance signaling pathways might be involved in regulating CST axon targeting.

Plexins belong to families of axon guidance molecules that act as receptors for semaphorin ligands. Together, they are by far the largest family of axon guidance molecules. Membrane-bound semaphorins (classes 4–7) directly interact with and signal through plexins, whereas most secreted semaphorins (class 3) signal through a receptor complex composed of plexins and their co-receptors, neuropilin (NPN)-1 or NPN-2 [18,19]. Semaphorin signaling through plexins is known to play roles in multiple aspects of neuronal development, and axon guidance is its most classical role [18-23]. Although several semaphorins have been shown to repel or attract neurites from cortical cultures *in vitro *[24-28], their roles in regulating the guidance of CST axons *in vivo *are still largely uncharacterized. Here we report that plexin (PLX)A3, PLXA4, and one of the membrane-bound semaphorins, Sema6A, are required for the dorsal turning of CST axons at the pyramidal decussation.

## Results

### The expression of *plexin-A3*, *plexin-A4*, and *neuropilin-1 *in cortical neurons coincides with the guidance of motor CST axons

To address whether semaphorin signaling through plexins regulates the guidance of CST axons, we focused on *PLXA3 *and *PLXA4*, as well as neuropilins, *NPN*-*1 *and *NPN*-*2*, and analyzed their expression patterns in the developing neocortex. The mRNAs of *PLXA3 *and *PLXA4 *were broadly expressed throughout the cortex from embryonic day (E) 18 to postnatal day (P) 0, immediately after layer V pyramidal neurons are born and migrate to their appropriate layer in the neocortex (Figure 1b–c", and data not shown). *NPN*-*1 *was also expressed in the developing neocortex at P0, but its expression was more restricted (Figure 1d–d"). By P3, once most CST axons have reached their targets in the spinal cord, *NPN*-*1 *expression in the cortex was reduced while *PLXA3 *and *PLXA4 *expression levels were maintained (data not shown). By contrast, *NPN*-*2 *transcripts were not expressed in the cortex during this time window (Figure 1e–e"). CST axons arise predominantly from type I layer V neurons [7,29], which specifically express a transcription factor, Ctip2 [30]. We found that a majority of Ctip2 immuno-positive pyramidal neurons co-expressed mRNA for *PLXA3*, *PLXA4*, and *NPN*-*1 *at P0 (Figure 1b'–d'). These results suggest that PLXA3, PLXA4, and NPN-1 play roles in guiding the developing motor CST axons to the spinal cord. To confirm their roles *in vivo*, we investigated whether the guidance of motor CST axons is affected in mutant mice lacking these genes.

### Plexin-A3 and plexin-A4 are required for dorsal turning of motor CST axons at the pyramidal decussation

A recent analysis of PLXA3 (PLXA3-/-) and PLXA4 mutant (PLXA4-/-) mice using NPN-1 expression as a marker suggested that NPN-1-positive axons projecting subcortically through the internal capsule and cerebral peduncles were defective in neonates [31]. To examine whether the initial guidance of CST axons through these structures is normal in PLXA3/PLXA4 double mutant (PLXA3/PLXA4-/-) mice, we studied the CST projections by using both L1-immunostaining at P1 [32] and biotinylated dextran amine (BDA) anterograde tracing of the motor CST axons at P25. Although subtle defects cannot be completely ruled out, targeting as well as fasciculation of these axons as they entered the internal capsule and cerebral peduncles appeared normal in P1 PLXA3/PLXA4-/- mice (n = 3) compared to wild-type (WT) mice (n = 3) (Figure 1f, g). When these initial projections from motor cortex were examined at P25 by BDA tracing, again the patterns were similar in WT (n = 3) and PLXA3/PLXA4-/- mice (n = 3) (Figure 1h, i), even though we could not exclude the possibility that subtle defects early on were corrected over time. Our results suggest that the CST projection through the internal capsule appears normal in PLXA3/PLXA4-/- mice.

**Figure 2:**
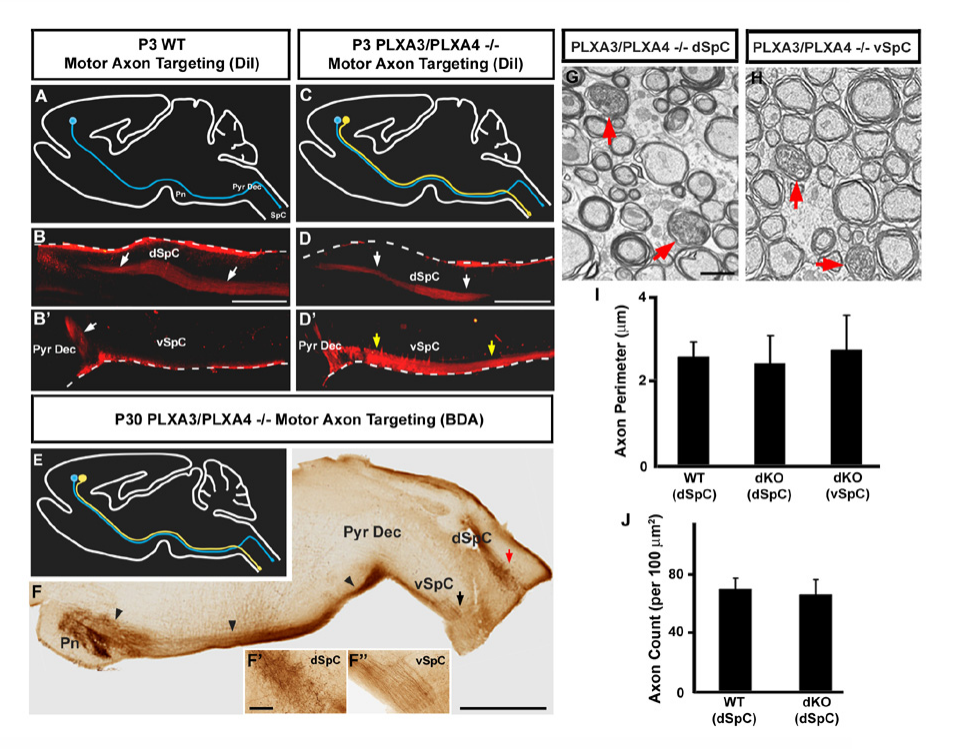
**Motor corticospinal axon pathfinding is abnormal in mice lacking PLXA3 and PLXA4.(a-b') **Diagram and sagittal view of the brain showing motor CST axons bilaterally labeled with DiI traveling within the pyramidal decussation (Pyr Dec; white arrow in (b')) and dorsal spinal cord (dSpC; white arrows in (b)) of P3 WT mice. **(c-d') **Diagram and sagittal view of the brain showing motor CST axons bilaterally labeled with DiI traveling past the pyramidal decussation and into the dorsal (white arrows in (d)) and ventral spinal cord (vSpC; yellow arrows in (d')) of P3 PLXA3/PLXA4-/- mice. Note that the white dashed lines in (b-b', d-d') indicate meninges surrounding the dorsal and ventral edges of the spinal cord and do not represent positive DiI labeling. **(e) **Diagram showing bilaterally labeled CST axons in P30 PLXA3/PLXA4-/- mice. In all diagrams (a, c, e), the normal motor CST axonal projection is indicated in blue and the abnormal ventral CST projection is indicated in yellow. **(f-f") **Course of BDA-labeled motor CST axons along the pyramidal tract in the brainstem (black arrowheads) of P30 PLXA3/PLXA4-/- mice. BDA-labeled axons were observed in the dorsal (dSpC; red arrow) and ventral (vSpC; black arrow) spinal cord. Higher power views of arrowed areas are shown in the insets of (f', f"). **(g, h) **Electron micrographs illustrating examples of BDA-labeled motor CST axons in the dorsal (g) and the ventrolateral (h) aspect of the cervical spinal cord in P30 PLXA3/PLXA4-/- mice. All labeled axons are myelinated (red arrows). **(i) **Average perimeters (mean ± standard error of the mean) of BDA-labeled axons are similar within the dorsal CST of P30 WT mice (n = 36 sections from 2 mice) and the dorsal (n = 13 sections from 2 mice) and ventrolateral (n = 26 sections from 2 mice) CST of P30 PLXA3/PLXA4-/- mice (dKO). *p *> 0.05, ANOVA, Neuman-Keuls test. **(j) **Average densities of axons (mean ± standard error of the mean of axons per 100 μm^2^) are similar in the dorsal CST of P30 WT (n = 36 sections from 2 mice) and PLXA3/PLXA4-/- mice (dKO; n = 13 sections from 2 mice). *p *> 0.05, Student's *t*-test. Each data set was averaged from randomly selected CST areas on all the electron micrographs taken from two animals. Scale bars: 500 μm (b-b', d-d'); 1,000 μm (f); 200 μm (f', f"); 1 μm (g, h).

We next examined the CST axons within the pyramidal tracts of the brainstem and the spinal cord by anterograde tracing. DiI (1,1'-dioctadecyl-3,3,3',3'-tetramethylindocarbocyanine perchlorate) or BDA tracers were bilaterally injected into the WT or mutant motor cortex to label the CST axonal projection down to the spinal cord. Although the guidance of motor CST axons through the brainstem structures was unaffected, we found a large DiI-labeled bundle of axons that diverged toward the ventrolateral aspect of the spinal cord at the pyramidal decussation in P3 PLXA3/PLXA4-/- mice (n = 4; Figure 2c–d'). By contrast, with this labeling technique, no abnormal ventral CST axons were observed in WT mice at P3 (n = 3; Figure 2a–b'). The abnormal ventrolateral CST persisted into adulthood in all PLXA3/PLXA4-/- mice (n = 14; Figure 2e–f"). Ultrastructural analysis of these mistargeted BDA-labeled axons demonstrated that they were myelinated and their perimeters were normal in size when compared with CST axons in WT mice (Figure 2g–i).

In the PLXA3/PLXA4-/- mutants, approximately one-half of the motor CST axons could still turn dorsally at the pyramidal decussation. These axons were properly guided across the midline and entered the dorsal funiculus in the spinal cord. This finding suggests that the turning is partially compensated for by other signaling *in vivo*. Alternatively, the partial defects in the plexin mutants may be due to the expression of PLXA3 and PLXA4 in a subset of CST axons. Given the broad expression patterns of these two genes (Figure 1b–c), it is more likely that additional molecules are required in the process. The properly guided CST axons in the spinal cord of PLXA3/PLXA4-/- mice remained well fasciculated as their axon densities were normal compared to WT mice (Figure 2j). In addition, we did not observe any errors in the targeting of transient motor CST axon collaterals to the superior colliculus in PLXA3/PLXA4-/- mice (n = 3) at P9 (data not shown). Taken together, these results indicate that signaling through PLXA3 and PLXA4 is utilized at the pyramidal decussation to control the dorsal turning of the motor CST axons *in vivo*.

### The abnormally guided CST axons in plexin-A3/plexin-A4 mutants do not cross the midline at the pyramidal decussation

**Figure 3:**
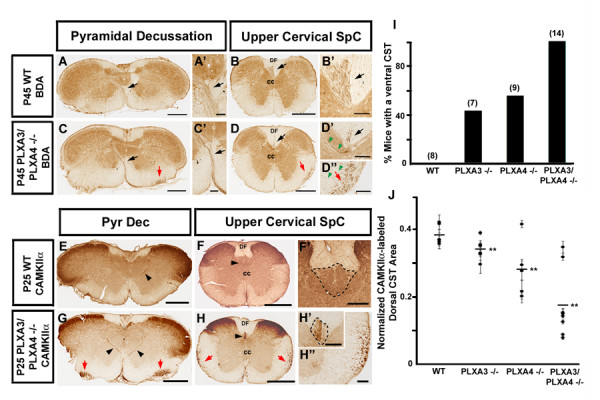
**Aberrant motor corticospinal axons in PLXA3 and PLXA4 double mutants are located in the ipsilateral, ventrolateral spinal cord.** All panels represent cross sections of the pyramidal decussation and spinal cord. Unilateral BDA motor CST axon tracing was performed in (a-d"). **(a, b) **Crossing of motor CST axons at the pyramidal decussation (Pyr Dec; black arrow in (a)) and into the dorsal funiculus of the spinal cord (SpC; black arrow in (b)) of P45 WT mice. **(c, d) **Normal crossed (black arrows) and aberrant uncrossed (red arrows) motor CST axons at the pyramidal decussation and spinal cord of P45 PLXA3/PLXA4-/- mice. **(a'-d") **High power views of arrowed areas in (a-d). In the upper cervical spinal cord, many mutant axons branched out from the uncrossed CST (green arrowheads in (d")). Some of these axons crossed to the contralateral gray matter (green arrowheads in (d')). **(e-h") **CamKIIα immunohistochemistry of the pyramidal decussation and spinal cord of P25 WT and P25 PLXA3/PLXA4-/- mice. Crossed (black arrowheads) and uncrossed (red arrows) CamKIIα-immunolabeled CST axons are observed at the pyramidal decussation and spinal cord in PLXA3/PLXA4-/-'s. High power views of the spinal cord in (f, h) are shown in (f', h', h"). Black dashed lines in (f', h') outline positive CamKIIα immunostaining of the dorsal CST in the dorsal funiculus of the spinal cord. **(i) **Comparison of percentages of 4- to 6-week old WT, PLXA3-/-, PLXA4-/-, and PLXA3/PLXA4-/- mice with an abnormal ventral CST apparent with BDA tracing. Numbers in parentheses indicate the number of mice analyzed. **(j) **Average normalized areas (see Materials and methods) of CamKIIα-labeled dorsal CST axons in WT, PLXA3-/-, PLXA4-/-, and PLXA3/PLXA4-/- mice. The dorsal CST area in each animal (mean ± standard error of the mean) is indicated by a black circle. The overall average dorsal CST areas (black lines) are decreased in the cervical spinal cords of PLXA3-/- (n = 6 mice), PLXA4-/- (n = 5 mice), and PLXA3/PLXA4-/- (n = 8 mice) versus WT (n = 6 mice) mice. **p < 0.01, Student's *t*-test. cc, central canal; DF, dorsal funiculus. Scale bars: 500 μm (a-h); 100 μm (a'-d", f'-h").

We also injected BDA unilaterally in the motor cortex to determine whether the abnormal ventrolateral spinal CST axons had crossed the midline. We examined the labeled CST axons in serial transverse sections and found that the abnormal CST axons maintained their course ipsilaterally at the pyramidal decussation and occupied a unique position in the ventrolateral region of the spinal cord in all PLXA3/PLXA4-/- mice (n = 6; Figure 3c–d"). Again, the ventrolateral axons were not seen in WT mice (n = 5; Figure 3a–b'). Consistent with the bilateral labeling results, some of the unilaterally labeled axons were found in the contralateral dorsal funiculus of PLXA3/PLXA4-/- mice, but the number was significantly reduced compared to WT mice (Figure 3b', d'). We also noticed that the ipsilateral ventrolateral CST axons in the mutant mice did not travel beyond the upper thoracic spinal cord. In these sections, many mutant axons could be seen branching from the ventrolateral CST and crossing to the gray matter of the contralateral dorsal spinal cord (Figure 3d', d"). This somewhat surprising observation suggests that at least some of the aberrant motor CST axons in PLXA3/PLXA4-/- mice can be directed to the appropriate final target area in the spinal cord.

To confirm that the misguided CST axons in mutant mice were motor axons, we labeled CST axons at P25 with an antibody against alpha calcium/calmodulin-dependent protein kinase type II (CamKIIα), which is specifically upregulated in motor CST axons in mice older than three weeks of age [33]. The results showed that the aberrant ventrolateral CST axons were labeled in bilateral regions of the medulla and spinal cord in PLXA3/PLXA4-/- (n = 4) but not WT (n = 3) mice (Figure 3e–h"), indicating that they are indeed CST motor axons. Since this marker stained all the motor axons, we also confirmed that the area of the dorsal funiculus occupied by CST axons in PLXA3/PLXA4-/- mice was considerably reduced compared to WT mice (Figure 3f', h', j).

We also assessed the individual contributions of PLXA3 and PLXA4 to the defect in single mutants. Although the phenotype was not present in all PLXA3-/- and PLXA4-/- mice, roughly equal numbers of PLXA3-/- and PLXA4-/- mice contained a ventrolateral CST (Figure 3i, and data not shown), suggesting that these two plexins partially compensate for each other's functions. Furthermore, we found that the area of the dorsal funiculus occupied by CST axons in PLXA3-/- and PLXA4-/- mice was smaller than in the WT, though the defect in the single mutants was less severe than in the PLXA3/PLXA4-/- animals (Figure 3j).

### Misguided ventrolateral CST axons are not observed in neuropilin mutants

**Figure 4:**
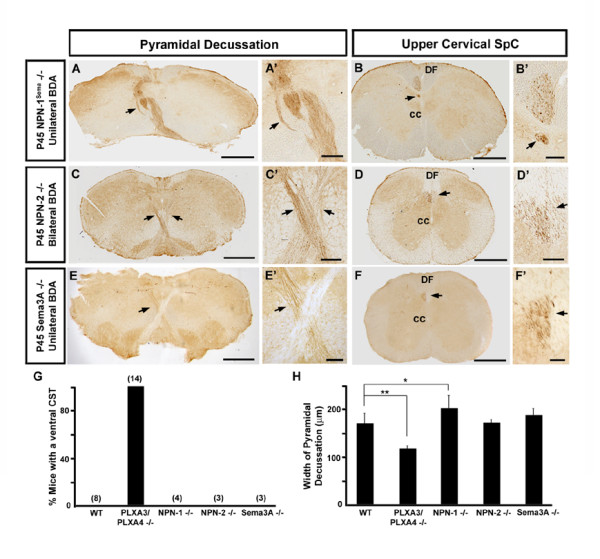
**Motor corticospinal axon turning at the pyramidal decussation is independent of neuropilins.(a-f) **Unilateral BDA motor CST axon tracing was performed in (a, b, e, f), and bilateral BDA motor CST axon tracing was performed in (c, d). **(a'-f') **Higher power views of arrowed areas in (a-f), respectively. The abnormal ventrolateral CST is not observed at the pyramidal decussation (a) and cervical spinal cord (SpC) (b) of P45 NPN-1^sema-/- ^mice. However, crossing motor CST fibers are noticeably defasciculated at the pyramidal decussation (black arrows in (a, a')) and the defasciculated axons form ectopic tracts in the contralateral spinal cord (black arrows in (b, b')). Motor CST axons in P45 NPN-2-/- (c, d) and Sema3A-/- (e, f) mice travel normally at the pyramidal decussation (black arrows in (c, c', e, e')) and cervical spinal cord (black arrows in (d, d', f, f')). **(g) **Comparison of percentages of WT and mutant mice with an abnormal ventral CST apparent with BDA tracing. Numbers in parentheses indicate the number of mice analyzed. This result indicates that in contrast to PLXA3/PLXA4-/- mice, there is no ventral CST in NPN-1-/-, NPN-2-/-, or Sema3A-/- mice. **(h) **Average width of the pyramidal decussation (mean ± standard error of the mean) in WT, PLXA3/PLXA4-/-, NPN-1-/-, NPN-2-/-, and Sema3A-/- mice. As expected, the width of the pyramidal decussation in PLXA3/PLXA4-/- mice (n = 6 mice) was smaller than WT mice (n = 5 mice). **p < 0.01, Student's *t*-test. In addition, the width of the pyramidal decussation was larger in NPN-1-/- mice (n = 4 mice) than WT, suggesting that CST axons are defasciculated in NPN-1-/- mice as they cross at the pyramidal decussation. *p < 0.05, Student's *t*-test. The width of the pyramidal decussation in NPN-2-/- mice (n = 2 mice) and Sema3A-/- mice (n = 4 mice) was similar to WT. cc, central canal; DF, dorsal funiculus. Scale bars: 500 μm (a-f); 100 μm (a'-f').

Our expression pattern studies predict that NPN-1, but not NPN-2, is required for the guidance of developing CST axons. To address whether neuropilins are required for targeting motor CST axons towards the contralateral dorsal spinal cord *in vivo*, we analyzed NPN-1^sema-/- ^(mutant mice expressing NPN-1 that lacks a semaphorin binding domain; n = 4) and NPN-2-/- (NPN-2 mutants; n = 3) for axon guidance defects in the CST axon projection [34,35]. As expected, no defects in motor CST axon guidance were observed in NPN-2-/- mice (Figure 4c–d', g–h). We did observe CST axon guidance defects in NPN-1^sema-/- ^mice, but the abnormality was qualitatively different from that seen in PLXA3/PLXA4-/- mice. All the CST axons from NPN-1^sema-/- ^mice turned dorsally and crossed the midline at the pyramidal decussation (Figure 4a, g). However, they were defasciculated when they crossed the midline and this resulted in a wider pyramidal decussation in NPN-1^sema-/- ^mice than in WT mice (Figure 4a', h). Some of these defasciculated axons formed ectopic tracts in the contralateral half of the dorsal spinal cord (Figure 4b'). As expected, we found that PLXA3/PLXA4-/- mice had a pyramidal decussation that was smaller in width than WT since only a subset of their axons crossed at the pyramidal decussation (Figure 4h). These data show that PLXA3/PLXA4 and NPN-1 are differentially required for CST axon guidance, and suggest that neuropilins and secreted (class 3) semaphorins are not involved in turning CST axons away from the ventral side of the pyramidal decussation.

To further support the conclusion that secreted semaphorins are not involved in CST axon turning at the pyramidal decussation, we examined the projections of motor CST axons in Sema3A (Sema3A-/-) and Sema3E mutant (Sema3E-/-) mice. Sema3A is expressed in the ventral spinal cord during development and has been thought to play a role in CST guidance by interacting with NPN-1 and L1 based on *in vitro *analyses [25,26]. In agreement with a recent report [36], we observed that the dorsal turning and midline crossing of motor CST axons at the pyramidal decussation was normal in Sema3A-/- mice (n = 3 BDA tracing, n = 2 CamKIIα immunostaining; Figure 4e–g). Further, in contrast to NPN-1^sema-/- ^mice, we found that the fasciculation of axons crossing at the pyramidal decussation was normal in Sema3A-/- mice (Figure 4h). Sema3E has recently been shown to bind directly to plexin [37]. We analyzed the expression pattern of Sema3E and found that Sema3E was not expressed in the ventral spinal cord. In accordance with this finding, we also found that the Sema3E-/- mice did not have a defect in CST axon guidance (n = 4; data not shown). Thus, our data support the role of PLXA3 and PLXA4 in CST axon turning at the pyramidal decussation that is independent of neuropilins and secreted semaphorins.

### Sema6A is required for proper guidance of motor CST axons

**Figure 5:**
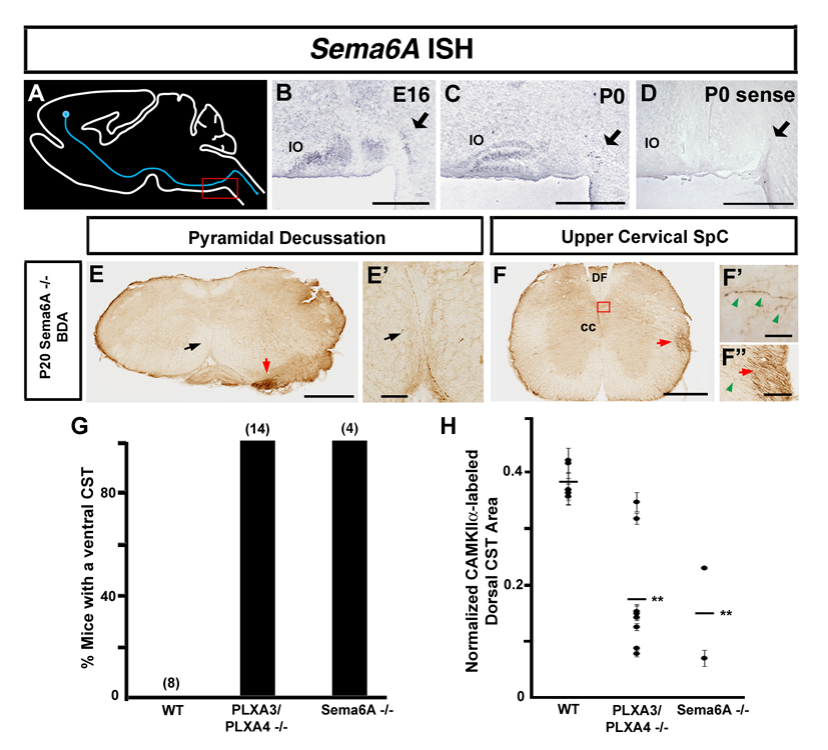
**Motor corticospinal axon turning at the pyramidal decussation requires Sema6A.(a-d) ***Sema6A *mRNA expression along the ventral pyramidal tract and pyramidal decussation (black arrows) during early motor CST axon guidance. Red box in (a) indicates the region of *Sema6A *expression shown in sagittal views of E16 (b) and P0 (c) WT mice, which is absent in the sense control (d). **(e, f) **Unilateral BDA motor CST axon tracing of P20 Sema6A-/- mice. **(e', f") **Higher power views of arrowed areas in (e, f), respectively. A boxed area in (f) is enlarged in (f'). Very few axons cross at the pyramidal decussation in Sema6A-/- mice (black arrows in (e, e')). Instead, axons form aberrant tracts (red arrows in (e, f, f")) in the ventrolateral spinal cord (SpC). Note that the aberrant tract moves out laterally as it traces down to the ispilateral spinal cord. The slightly different locations of the ectopic ventrolateral tracts seen here as compared to those seen in the PLXA3/PLXA4-/- mice in Figure 3 are due to different rostrocaudal locations of the sections. Similar to that seen in PLXA3/PLXA4-/- mice, many of these ventrolateral axons branch back toward the contralateral dorsal cervical spinal cord, though they are mistargeted below the dorsal funiculus (green arrowheads in (f', f")). **(g) **Comparison of percentages of WT, PLXA3/PLXA4-/-, and Sema6A-/- mice with an abnormal ventral CST apparent with BDA tracing. Numbers in parentheses indicate the number of mice analyzed. **(h) **Average normalized areas (see Materials and methods) of CamKIIα-labeled dorsal CST axons in WT, PLXA3/PLXA4-/-, and Sema6A-/- mice. The dorsal CST area in each animal (mean ± standard error of the mean) is indicated by a black circle. The overall average dorsal CST area (black lines) is decreased in the cervical spinal cords of Sema6A-/- (n = 2 mice) versus WT (n = 6 mice) mice. **p < 0.01, Student's *t*-test. cc, central canal; DF, dorsal funiculus, IO, inferior olive. Scale bars: 500 μm (b-f); 100 μm (e'f"); 25 μm (f').

To explore the possible semaphorin cue(s) that activate PLXA3/PLXA4 signaling to guide the CST axons dorsally at the pyramidal decussation, we turned to membrane-bound semaphorins. Since PLXA4 is known to interact with Sema6A in a neuropilin-independent manner [38,39], we studied the expression pattern of *Sema6A *and analyzed the targeting of motor CST axons in Sema6A mutant (Sema6A-/-) mice. We found that *Sema6A *was expressed ventrally along the posterior pyramidal tract and pyramidal decussation between E16 and E18 (Figure 5a–b; and data not shown). By P0, when the majority of the motor CST axons have crossed the pyramidal decussation into the dorsal spinal cord, *Sema6A *expression was restricted to the inferior olive and the pyramidal decussation, though the latter appeared to be less prominent than at earlier stages (Figure 5c). This expression pattern suggested that Sema6A could be the ligand responsible for the plexin-mediated dorsal turning of motor axons at the pyramidal decussation. In Sema6A-/- mice (n = 4), we observed mistargeted axons in the ventrolateral spinal cord using anterograde BDA tracing similar to what was seen in PLXA3/PLXA4-/- mice (Figure 5e, g). However, the defect appeared to be more severe because relatively fewer labeled Sema6A-/- axons turned dorsally at the pyramidal decussation (Figure 5e'). In addition, the variation of defects from animal to animal was relatively broad such that each animal had fairly varied numbers of axons that crossed at the pyramidal decussation, but the majority of these animals appeared to have a more severe defect than the PLXA3/PLXA4-/- mice (Figures 3c–d" and 5e–f", and data not shown). We further assessed the severity of the defect in Sema6A-/- mice with CamKIIα staining (n = 2) and found that the defect in these animals was very similar to that of the PLXA3/PLXA4-/- mice (Figure 5h). As noted in the PLXA3/PLXA4-/- mice, the misguided ventrolateral CST axons branched out and targeted to the contralateral gray matter at the level of the cervical spinal cord (Figure 5f–f"). These analyses indicate that membrane-bound Sema6A is one of the local cues that induces proper turning of motor CST axons dorsally at the pyramidal decussation.

## Discussion

The development of the CST has served as a classic example for studying the guidance of long-range axons [7,9]. In the CNS, midline-crossing is an important phenomenon for the guidance of long axons [40,41]. During development, the ventrally positioned CST axons make dorsal turns to cross the midline at the pyramidal decussation. Previous reports have indicated that multiple signaling systems are utilized to ensure the dorsal turning and midline crossing of CST axons at the pyramidal decussation [10]. These include the netrin/DCC/Unc5h signaling system and the Ig superfamily signaling system. We report here that the semaphorin/plexin signaling system is also involved in guiding CST axons dorsally at the pyramidal decussation.

By comparing the reported CST defects in mutant mice from these signaling systems, we find that they may function in a cooperative fashion to regulate the guidance of CST axons at the pyramidal decussation. However, major phenotypical differences are also noted between different systems. In the netrin/DCC/Unc5h signaling system [12], netrin is expressed at the midline beneath the central canal at the point at which CST axons decussate. DCC and Unc5h are netrin receptors responsible for axon attraction and repulsion, respectively. In DCC mutants, CST axons are not attracted by the midline netrin signal so the axons do not make the dorsal turn at the decussation and all the CST axons remain within the ventral spinal cord. In Unc5h3 mutants, some CST axons stay ventrolaterally, whereas others can turn dorsally and cross the midline. However, in contrast to what we have observed in PLXA3/PLXA4-/- mice, those Unc5h3 mutant axons that cross the midline do not target the dorsal funiculus, but enter the dorsal gray matter instead. Thus, the netrin/DCC/Unc5h signaling system seems to mainly control the dorsal turning of CST axons at the pyramidal decussation and the proper targeting of CST axons to the dorsal funiculus.

The roles of the Ig superfamily signaling system in regulating the dorsal turning and midline crossing of CST axons are diverse [42]. In young NCAM mutant mice [14], many CST axons fail to turn dorsally and remain in the ventrolateral spinal cord. Among the mutant axons that make the dorsal turn at the pyramidal decussation, many fail to cross the midline and instead project to the ipsilateral dorsal funiculus. However, the abnormal CST axons are absent in adult NCAM mice, suggesting either a correction or loss of aberrant fibers. In adult L1 mutants [13], all CST axons turn dorsally at the pyramidal decussation, but many of them stay ipsilateral as they project to the dorsal funiculus. Therefore, the Ig superfamily signaling system seems to control both dorsal turning and midline crossing of the CST axons. It is interesting to note that the L1 subfamily of Ig molecules, including L1, NrCAM, and CHL1, also interact with neuropilins to mediate the signals from secreted semaphorins [25,26,43,44]. *In vitro *evidence has suggested that Sema3A signaling through an L1/NPN-1 complex contributes to midline crossing of CST axons at the pyramidal decussation [25]. However, *in vivo *analysis of the Sema3A-/- mouse by our lab and others [36] indicates no defects in dorsal turning or midline crossing of the CST in this mutant. We also show that, in contrast to L1 mutant mice, all the CST axons cross the midline in NPN-1-/- mice even though they are defasciculated. These results suggest NPN-1 and L1 function independently in regulating CST guidance at the pyramidal decussation. Recently, CHL1 has been shown to function together with NPN-1 to mediate the guidance of thalamocortical axons *in vivo *[44]. It would be interesting to test whether CHL1 is also involved in CST axon guidance.

**Figure 6:**
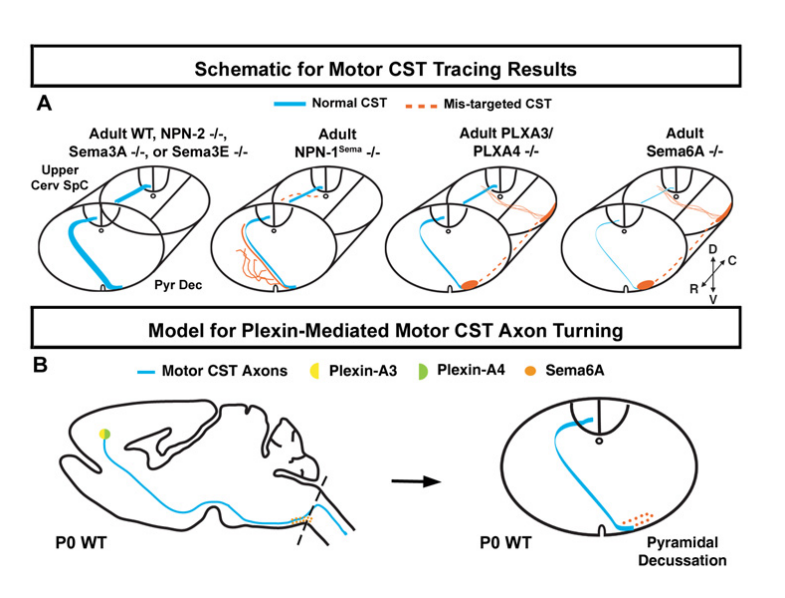
**Summary of CST axon guidance in WT and knockout animals and a model for plexin signaling in CST axon turning. (a) **Diagrams of cross sections of brainstem and spinal cord summarizing CST axon guidance defects observed in adult mutant mice. Ventrolateral CST axons were only observed in plexin and Sema6A mutants. The Sema6A-/- phenotype was relatively diverse between animals. Summarized in this diagram are the major defects. **(b) **Model for motor CST axon turning at the pyramidal decussation (refer to Discussion). C, caudal; D, dorsal; Pyr Dec, pyramidal decussation; R, rostral; SpC, spinal cord; V, ventral.

Our analysis has revealed the contributions of semaphorin/plexin signaling in the dorsal turning of motor CST axons at the pyramidal decussation (Figure 6). Specifically, we demonstrate that in the absence of PLXA3 and PLXA4, up to 50% of the motor CST axons are guided to the ventral spinal cord, resulting in an abnormal ipsilateral ventrolateral tract. The plexin-mediated CST turning defect appears to be neuropilin-independent as NPN-1-/- and NPN-2-/- mice do not display ventrolateral CST guidance defects. We also found that neither Sema3A-/- nor Sema3E-/- mice had such defects. These results indicate that the local environmental cues that act at the pyramidal decussation to direct plexin-mediated dorsal turning of motor CST axons are membrane-bound semaphorins. In support of this, we found that Sema6A-/- mice had a similar motor CST guidance defect to PLXA3/PLXA4-/- mice in which the majority of axons stayed ipsilateral and formed a ventrolateral tract.

Several recent reports have nicely addressed the interactions between class 6 semaphorins and plexin-A family members. Specifically, it has been shown that PLXA4 directly binds Sema6A, and their interactions *in vivo *are important for the lamina-specific projection of mossy fibers in the hippocampus [39] and for the short-range repulsion of developing sympathetic axons [38]. In addition, it has been shown that Sema6A binds PLXA2 [45], which is also expressed in the motor cortex during CST axon guidance (data not shown). However, analysis of the PLXA2 mutant mouse revealed no defects in CST axon guidance (KJ Mitchell, personal communication). We have previously shown that PLXA3 and PLXA4 are co-expressed in neuronal tissues to mediate axon repulsion, axon pruning and neuronal migration [46-49], but these functions are mostly activated by secreted semaphorins. Here, our phenotypic analysis in mutant mice suggests that PLXA3 and PLXA4 may function with membrane-bound Sema6A *in vivo*. However, it is still unclear whether PLXA3 can directly bind to Sema6A, and how PLXA3 and PLXA4 interact to mediate Sema6A signals. It is also important to note that the CST guidance defects in Sema6A-/- mice are apparently more diverse and more severe than in PLXA3/PLXA4-/- mice. Although no apparent CST guidance defects were noted before axons reached the hindbrain in PLXA3/PLXA4-/- mice, guidance defects have been noted at the midbrain-hindbrain boundary in Sema6A-/- mice (KJ Mitchell, personal communication). This defect higher up in the CST projection pathway may account for the more severe defect in CST guidance across the pyramidal decussation seen in some Sema6A-/- mice. It is now apparent that CST axons are guided by specific signals at different choice points to reach their distant targets. The phenotypic differences between Sema6A-/- and PLXA3/PLXA4-/- mice indicate that other plexin or non-plexin receptors may also be used to mediate Sema6A signals in the guidance of motor CST axons.

## Conclusion

We have characterized the roles of PLXA3, PLXA4, NPN-1, NPN-2, Sema3A, Sema3E, and Sema6A in regulating the guidance of motor CST axons to the dorsal spinal cord *in vivo *(summarized in Figure 6). We find that PLXA3, PLXA4, and Sema6A are required for the proper dorsal turning of motor CST axons at the pyramidal decussation. As motor CST axons are crossing the midline, we find that NPN-1 is required for CST axons to remain fasciculated so they may target the dorsal funiculus appropriately. However, PLXA3 and PLXA4 are either compensated for by other receptors in this process or not required. We also find that the dorsal turning and midline crossing of motor CST axons are normal in NPN-2, Sema3A, and Sema3E mutants. Although many questions remain, it is evident that semaphorin signaling is one of several signaling systems that coordinate at specific points along the pathway to properly guide the long CST axons from the cerebral cortex to the spinal cord.

## Materials and methods

### Mouse breeding

Animal protocols were approved by the Institutional Animal Care and Use Committee at UC Davis. Genotyping on knockout mice was carried out as described previously [34,35,49-52]. NPN-1^sema-/- ^mice were obtained from Jackson Laboratories (Bar Harbor, ME, USA). Sema3A-/- mice were a generous gift from Mark Fishman and Marc Tessier-Lavigne. Sema3E-/- and Sema6A-/- mice were a generous gift from Marc Tessier-Lavigne.

### Mouse tracer injections

Wild-type and mutant mice were injected with various tracers at different postnatal ages (P0 to P45). DiI (Molecular Probes, Carlsbad, CA, USA) and BDA (Molecular Probes) anterograde tracing was performed as described previously [53,54]. Mice were injected blindly prior to determining genotype. Briefly, DiI (20% in N,N-dimethylformamide) or BDA (10–20% in phosphate buffered saline) were injected focally in the motor cortex of WT and mutant mice *in vivo *and allowed to trace for a minimum of three days. Locations of the injection sites were confirmed in sagittal sections of the cortex to ensure tracers were injected in the appropriate regions of the cortex.

### Immunohistochemistry, *in situ *hybridization, and EM processing

Immunohistochemistry was performed on floating sections as described previously [55]. Antibodies and concentrations used in the study were: CamKIIα (1:1,000; Chemicon, Temecula, CA, USA), Ctip2 (1:1,000; Abcam, Cambridge, MA, USA), and L1 (1:1,000; Chemicon). The plexin and neuropilin probes for *in situ *hybridization and the procedures for radioactive α-^33^P *in situ *hybridization were as described previously [51,56]. The procedure for non-radioactive *in situ *hybridization was as described previously [51]. Sections that contained BDA-labeled CST axons were preserved for ultrastructural analysis with EM as described [55].

### Analysis of CamKIIα immunostained spinal cord sections

Transverse sections of CamKIIα-immunostained at the level of the pyramidal decussation or cervical spinal cord were selected for analysis. Raw images of the sections were digitally captured with a CCD camera (Zeiss, Thornwood, NY, USA) and imported into PhotoShop (Adobe Systems, San Jose, CA, USA). For quantification of CST area in the dorsal funiciulus, images were cropped and only the dorsal funiculus area was preserved for further analysis. Grayscaled images were thresholded to 30% above background levels as described [57]. Pixels that were above threshold were considered as positive labeling and these areas were measured using Image J (NIH, Bethesda, MD, USA). Positively labeled areas were subsequently normalized to the total area of the dorsal funiculus. For quantification of fasciculation at the pyramidal decussation, the width of the pyramidal decussation was measured in all available brainstem sections containing it.

Statistics for all data were obtained from Statistica 6.0 (Statsoft, Tulsa, OK, USA) or Microsoft Excel with a Benjamini and Hochberg correction for multiple comparisons.

## Abbreviations

BDA: Biotinylated dextran amine; CamKIIα: alpha calcium/calmodulin-dependent protein kinase type II; CNS: Central nervous system; CST: Corticospinal tract; DiI: 1,1'-dioctadecyl-3,3,3',3'-tetramethylindocarbocyanine perchlorate; E: Embryonic day; Ig: Immunoglobulin; NPN: Neuropilin; P: Postnatal day; PLX: plexin; WT: Wild type.

## Competing interests

The authors declare that they have no competing interests.

## Authors' contributions

LKL and HJC initiated the project. RLF, LKL, XBL, EGJ, and HJC discussed and designed the experiments. RLF, LKL, XBL, and JC performed the experiments and analyzed the data. RLF, LKL, and HJC wrote the paper.
